# Is the patella apprehension test a valid diagnostic test for patellar instability? A systematic review

**DOI:** 10.1016/j.jor.2023.07.005

**Published:** 2023-07-13

**Authors:** Diego Agustín Abelleyra Lastoria, Bethany Kenny, Sara Dardak, Charlotte Brookes, Caroline Blanca Hing

**Affiliations:** a St George's University London, St George's University Hospitals NHS Foundation Trust, London, SW17 0RE, United Kingdom; b Department of Trauma and Orthopaedics, St George's University Hospitals NHS Foundation Trust, London, SW17 0RE, United Kingdom; c Department of Trauma and Orthopaedics, Queen Elizabeth the Queen Mother Hospital, Margate, CT9 4AN, United Kingdom

**Keywords:** Patella apprehension test, Patellar instability, Systematic review

## Abstract

**Introduction:**

Patellar instability can arise from a traumatic event with anatomical predisposing factors increasing the risk of dislocation. Accurate diagnosis is required to initiate appropriate treatment. We aimed to evaluate the patella apprehension test (PAT) as a method to diagnose patellar instability.

**Methods:**

The PRISMA diagnostic test accuracy checklist was followed. The review protocol was registered on PROSPERO with registration number CRD42022357898. Electronic databases, currently registered studies, conference proceedings and the reference lists of included studies were searched. A narrative synthesis evaluated the validity of the PAT as a method of diagnosing patellar instability.

**Results:**

A total of 4867 records were screened in the initial search. Of these, 34 articles satisfied the inclusion criteria, assessing 1139 knees of 1046 patients. The PAT was found to have a high sensitivity and specificity. Its intra and inter-rater reliability was highly variable among studies. Studies reporting patellar instability correction following surgery also found a decrease in the number of patients exhibiting a positive PAT.

**Conclusion:**

Current evidence suggests that the PAT has a high sensitivity and specificity. The intra- and inter-rater validities of the PAT are widely variable due to its subjective nature. Thus, though the PAT can be used to provide a provisional clinical diagnosis of patellar instability, formal functional assessment and imaging should be performed to confirm the diagnosis. Further research should explore the association between a positive PAT and anatomical parameters. In addition, studies comparing the accuracy of the PAT and radiological investigations should be performed.

## Abbreviations

PAT: patella apprehension test
MPFL: medial patellofemoral ligament
CT: computerized tomography
EFORT: European federation of national associations of Orthopaedics and traumatology
AQUA: anatomical quality assessment
MPFLR: medial patellofemoral ligament reconstruction
MPAT: modified version of the patella apprehension test
RDPAT: reversed dynamic patella apprehension test
TT-TG: tibial tuberosity – tibial groove
TT – PCL: tibial tuberosity – posterior cruciate ligament
VAS: visual analogue scale
BPII 2.0: Banff Patellofemoral Instability Score 2.0

## 1 Introduction

Patellar instability can lead to patellar dislocation, which accounts for 2–3% of injuries of the knee joint.^1^ Its incidence is 6 in every 100,000 patients per year.^2^ This can lead to femoral condyle contusion, knee effusion, and rupture of the medial patellofemoral ligament (MPFL).^1^^,^^3^ The latter can result in tearing of the medial retinaculum.^1^ Recurrent dislocations occur in 15%–44% of patients who experience an initial patellar dislocation.^4^ In addition, 58% of patients note limitations when carrying out strenuous activity six months after the injury. Other symptoms include severe knee pain, swelling, and the inability to run.^5^

The PAT is used in clinical practice to diagnose patella instability. It was first described by Ferrari et al., who performed the PAT with the patient standing.^6^ The test is considered positive if the patient reports apprehension or instability during internal rotation of the torso. However, in current clinical practice, the PAT is more commonly performed with the patient lying supine. Firm pressure is applied to the medial border of the patella in an extended and relaxed knee. A positive finding occurs when the patient expresses apprehension that the patella will dislocate,^7^ with instability or discomfort being reported.^8^ A correct identification of patellar instability is required to initiate any of the appropriate treatment strategies. These range from conservative approaches like closed reduction and rehabilitation, to surgical interventions like lateral retinacular release and osteotomy.^7^

Adequate identification of patellar instability through physical examination is required, since radiological studies may be insensitive in the non-acute setting. Radiographs and computerized tomography (CT) scans are static, and may not help visualize a dislocated patella.^9^ The PAT is widely used in clinical practice. Considering this, its validity should be formally assessed to determine whether its common use is justified. A previous review, conducted in 2008, revealed the validity of the PAT was unclear.^10^ Articles published since the previous study were therefore evaluated to determine an accurate and updated assessment of the validity of the PAT. The aim of this systematic review was therefore to determine whether the PAT is a valid way of diagnosing patellar instability.

## 2 Methods

We aimed to determine whether the PAT is a valid way of diagnosing patellar instability. The PRISMA diagnostic test accuracy checklist was followed.^11^ The protocol for this review was registered on PROSPERO with registration number CRD42022357898.

### 2.1 Study eligibility

Study eligibility was determined by following the pre-specified criteria. All studies reporting on results of the PAT both before and after intervention (conservative or surgical) were included, as well as those assessing its validity or reliability. Papers not reporting original data such as literature or systematic reviews were excluded, along with case reports, animal studies, cadaveric studies and letters to the editor. Studies describing theoretical models, studies not reporting on PAT outcomes, and studies in which the PAT was not performed pre- and post-operatively were also excluded. There were no language or publication status constraints.

### 2.2 Search strategy and data extraction

We searched the following electronic databases from their inception to 06/09/2022: MEDLINE, Embase, CINAHL, the Cochrane Library, PEDro, and AMED. Study registries, including the ISRCTN registry, the UK National Research Register Archive, the WHO International Clinical Trials Registry Platform, OpenSIGLE, and the National Institute for Health Research Portfolio were reviewed. Conference proceedings from the European federation of national associations of Orthopaedics and traumatology (EFORT), British Orthopaedic Association and British Trauma Society were searched. The reference lists of included studies were also searched.

Database search was conducted independently by three reviewers. Reviewers screened records independently. Disagreements regarding study eligibility were solved by discussion. The first search was first conducted on 17/11/2021, and repeated on 06/09/2022. The search strategy is attached (Appendix A). Data extraction was conducted by the first author.

### 2.3 Methodological appraisal

Two reviewers independently evaluated the level of evidence and risk of bias of each study. The level of evidence of the studies presented was determined with the Oxford CEBM: Levels of Evidence.^12^ The Cochrane Collaboration's tool for Non-Randomized Studies was used to perform a risk of bias assessment for non-randomized interventional studies.^13^ The IHE case series quality appraisal checklist was used to assess risk of bias in interventional case series.^14^ The AQUA tool was used to assess risk of bias in non-interventional anatomical studies.^15^

### 2.4 Data synthesis strategy

Quantitative pooled analysis was prevented by the heterogeneity of the data in terms of interventions performed, outcomes evaluated, and approach to the PAT. Therefore, a narrative synthesis was performed. Number of patients and knees, follow-up duration, mean patient age. and degree of patellar instability were extracted. In addition, intervention performed, stability scores, pre- and post-operative PAT (positive or negative), and stability outcomes were presented in Table 2.

## 3 Results

**Fig. 1:**
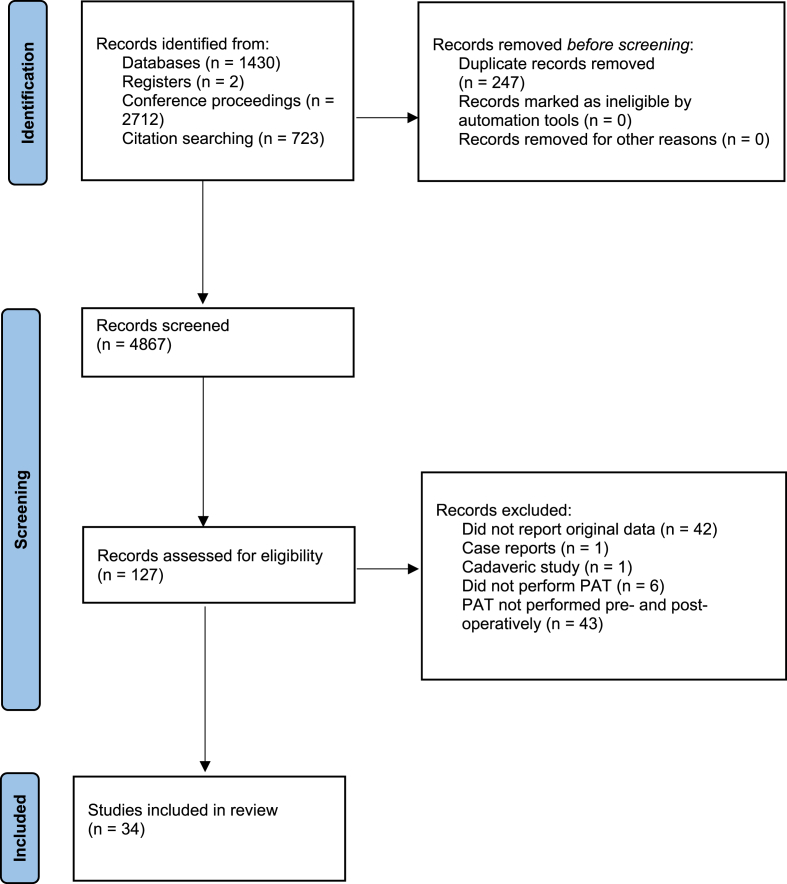
PRISMA diagram depicting the study collection process.

A total of 4867 records were screened, with 127 potentially eligible articles identified (Fig. 1). Ninety-three articles were excluded based on the pre-specified exclusion criteria. Thirty-four studies were included, evaluating 1139 knees of 1046 patients. Table 1 depicts subjects’ baseline characteristics. Thirty studies reported on patients with recurrent patellar instability (2 or more episodes). In two studies it was unclear whether patellar instability was recurrent or acute.^16^^,^^17^ Ahmad et al. used the PAT to diagnose instability in patients for which this was suspected.^9^ Mochizuki et al. did not report outcomes separately for patients with first time and recurrent patellar dislocations.^18^

**Table 1:** Level of evidence, risk of bias, and baseline characteristics of studies included.

Study	Study design, level of evidence	Risk of bias	Number of patients (males, females)	Number of knees	Mean patient age (years)	Follow-up duration
Malecki et al., 2016^28^	Case control, 3	Some concerns	56 (12, 44)	Group 1: 32	14	5.6 years
				Group 2: 33		
Leite et al., 2021^32^	Case series, 4	Some concerns	25 (3, 22)	31	28.7	2.62 years
Sadigursky et al., 2017^33^	Case series, 4	Some concerns	7 (4, 3)	7	11.28	1 year
Akgün et al., 2010^20^	Case series, 4	NFT	16 (11, 5)	17	25	2.6 years
Ahmad et al., 2009^9^	Non-interventional anatomical study, 2b	Low	51 (24, 27)	51	24.2	NA
Hiemstra et al., 2021^31^	Case series, 4	Some concerns	38 (11, 27)	76	24.7	NA
Zimmermann et al., 2019^30^	Non-interventional anatomical study, 2b	Low	RDPAT: 78 (35, 43)	113	RDPAT: 22	NA
			Control: 35 (16, 19)		Control: 31	
Wang et al., 2010^34^	Case series, 4	Some concerns	Group 1: 28 (10, 18)	69	Group 1: 29	3.5 years
			Group 2: 41 (20, 21)		Group 2: 31	
Torkaman et al., 2015^35^	Case series, 4	Some concerns	15 (6, 9)	15	26.5	1 year
Xu and Zhao, 2011^21^	Case series, 4	NFT	28 (5, 23)	28	14.7	4.8 years
Ma et al., 2012^16^	Cohort study, 2b	Some concerns	Group 1: 29 (13, 16)	Group 1: 29	Group 1: 13	50 months
			Group 2: 32 (12, 20)	Group 2: 32	Group 2: 14	
Fink et al., 2014^36^	Case series, 4	Some concerns	17 (7, 10)	17	21.5	12 months
Smith et al., 2012^25^	Case series, 4	Low	5 (0, 5)	10	26.6	NA
Song et al., 2014^37^	Case series, 4	Some concerns	20 (10, 10)	20	21	34.5 months
Ellera Gomes et al., 2004^38^	Case series, 4	Some concerns	15 (4, 11)	16	26.7	5 years
Ntagiopoulos et al., 2013^39^	Case series, 4	Some concerns	27 (14, 13)	31	21	7 years
Beckert et al., 2016^40^	Case series, 4	Some concerns	17	19 (18, 1)	29.5	2 years
Calanna et al., 2016^41^	Case series, 4	Some concerns	19 (12, 7)	19	25.5	22 months
Fadel and Hosni, 2020^27^	Case series, 4	Low	34 (5, 29)	34	19.4	28.7 months
Yang et al., 2017^23^	Case series, 4	NFT	12	12	NR	16.4 months
Watanabe et al., 2008^19^	Case series, 4	NFT	40	Group 1: 29	NR	4.3 years
				Group 2: 13		
Elbarbary et al., 2020^42^	Case series, 4	Some concerns	7 (2, 5)	7	23.7	18.8 months
Kang et al., 2014^43^	Case series, 4	Some concerns	45 (18, 27)	45	26.6	33.7 months
Niu et al., 2017^26^	Case series, 4	Low	30 (10, 20)	30	25	4 years
Chen et al., 2015^22^	Case series, 4	NFT	28 (4, 24)	28	21.8	41.8 months
Li et al., 2018^17^	Case series, 4	NFT	28 (6, 22)	32	21	6 months
Camathias et al., 2016^44^	Case series, 4	Some concerns	44 (14, 30)	50	15.6	2 years
Mochizuki et al., 2019^18^	Case series, 4	Some concerns	24 (6, 18)	24	25.4	2 years
Mahmoud et al., 2021^45^	Case series, 4	Some concerns	10	10	26	19.4 months
Kumahashi et al., 2016^46^	Cohort study, 2b	Some concerns	15 (3, 12)	17	22	45 months
Hiemstra et al., 2021^24^	Case series, 4	High	89	98	NR	2 years
Kumahashi et al., 2012^29^	Case series, 4	Some concerns	5 (2, 3)	5	13.6	27.8 months
Schöttle et al. 2005^47^	Case series, 4	Some concerns	12 (4, 8)	15	30.1	47 months
Kita et al., 2012^48^	Case series, 4	Some concerns	24 (6, 18)	25	22.7	13.2 months

**Table 2:** Stability and PAT outcomes following different surgical interventions.

Study	Intervention	Pre-operative patella stability score	Post-operative patella stability score	Pre-operative PAT	Post-operative PAT	Stability outcomes
Malecki et al., 2016^28^	Group 1: MPFL reconstruction Group 2: Combination of: retinacular plasty, vastus medialis advancement, and Roux-Goldthwait procedure	NR	Group 1: Kujala score: 90.8 Lysholm score: 89.8 Group 2: Kujala score: 85 Lysholm score: 84.2	All patients had a positive PAT pre-operatively	6 patients in group 1 (19%) and 6 in group 2 (18%) noted a positive PAT	Group 1: 3 dislocations noted Group 2: 4 dislocations noted
Leite et al., 2021^32^	Tibial tubercle osteotomy with concomitant distalization and MPFL reconstruction	Kujala score: 52	Kujala score: 77	All patients had a positive PAT pre-operatively	All patients had a negative PAT pre-operatively	J-sign improved in 30 cases (97%). One case (3%) of recurrent instability was reported
Sadigursky et al., 2017^33^	MPFL and medial patellotibial ligament reconstruction	Kujala score: 42.57 Lysholm score: 33.71	Kujala score: 88.57 Lysholm score: 87.71	All patients had a positive PAT pre-operatively	All patients had a negative PAT post-operatively	J-sign was noted in 6 patients pre-operatively (85.7%), compared to 1 post-operatively (14.3%)
Akgün et al., 2010^20^	Fulkerson osteotomy	NR	Kujala score: 82.6	All patients had a positive PAT pre-operatively	PAT was negative in 14 knees (82.4%), and positive in three (17.6%)	Subluxation was noted in 2 knees (11.8%). Patellar tilt and subluxation were corrected in all but two knees.
Wang et al., 2010^49^	Group 1 (MPFLR): 28 Group 2 (MPFLR and vastus medialis advancement): 41	Kujala score: Group 1: 51.3 Group 2: 53.7	Kujala score: Group 1: 79.9 Group 2: 83.9	All patients had a positive PAT pre-operatively	Group 1: 20 had a negative PAT (71.4%) Group 2: 38 had a negative PAT (92.7%)	Recurrent dislocation was not reported in any patient post-operatively
Torkaman et al., 2015^35^	MPFLR	Kujala score: 59.8	Kujala score: 88.6	All patients had a positive PAT pre-operatively	All patients had a negative PAT post-operatively	NR
Xu and Zhao, 2011^21^	Arthroscopic medial retinaculum plication	Kujala score: 66.7 Lysholm score: 52.6	Kujala score: 76.6 Lysholm score: 70.7	All patients had a positive PAT pre-operatively.	At 2 years follow-up, 12 demonstrated a positive PAT (42.9%).	Six patients suffered from redislocation (21.4%), and 23 patients experienced patella instability (82.1%).
Ma et al., 2012^16^	Group 1: Medial capsule reefing Group 2: Medial patellar retinaculum plasty	Kujala score: Group 1: 52.3 Group 2: 53.5	Kujala score: Group 1: 78.1 Group 2: 82.2	All patients had a positive PAT pre-operatively	Group 1: 20 patients had a negative PAT (68.9%) Group 2: 30 patients had a negative PAT (93.8%)	Medial retinacular plasty was better than medial capsule reefing in decreasing the rate of patellar dislocation
Fink et al., 2014^36^	MPFLR	Kujala score: NR Lysholm score: 69.5	Kujala score: 89.2 Lysholm score: 88.1	All patients had a positive PAT pre-operatively	Fifteen patients had a negative PAT (88.2%)	No dislocation during the follow-up period
Song et al., 2014^37^	MPFLR	Kujala score: 52.6 Lysholm score: 49.2	Kujala score: 90.9 Lysholm score: 90.9	All patients had a positive PAT pre-operatively	Nineteen patients had a negative PAT (95%)	No patient experience a redislocation
Ellera Gomes et al., 2004^38^	MPFLR	NR	NR	All patients had a positive PAT pre-operatively	Fifteen patients had a negative PAT (93.8%)	Patellar tracking was normal in 14 knees (87.5%)
Ntagiopoulos et al., 2013^39^	Sulcus-deepening trochleoplasty	Kujala score: 59	Kujala score: 87	PAT was positive in 30 knees (96.7%)	PAT was positive in 6 knees (19.4%) PAT was negative in 25 knees (80.6%)	No recurrence was observed
Beckert et al., 2016^40^	Lateral patellofemoral ligament reconstruction	NR	NR	All patients had a positive PAT pre-operatively	All patients had a negative PAT post-operatively	No recurrence of patellar instability was observed
Calanna et al., 2016^41^	MPFLR	Kujala score: 65.2 Lysholm score: 64.3	Kujala score: 94.7 Lysholm score: 94.7	PAT was positive in 17 patients (89%)	PAT was positive in 2 cases (11%)	No recurrence of patellar dislocation was reported
Fadel and Hosni, 2020^27^	MPFLR	Kujala score: 69.5	Kujala score: 94.8	All patients had a positive PAT pre-operatively	All patients had a negative PAT post-operatively	No recurrence of patellar dislocation was reported
Yang et al., 2017^23^	MPFLR	NR	NR	All patients had a positive PAT pre-operatively	All patients had a negative PAT post-operatively	No recurrence of patellar dislocation was reported
Watanabe et al., 2008^19^	Group 1: MPFLR Group 2: MPFLR + tibial tubercle transfer	Lysholm score: Group 1: 70 Group 2: 72	Lysholm score: Group 1: 92 Group 2: 90	Group 1: 28 had a positive PAT (97%) Group 2: 11 had a positive PAT (92%)	Group 1: 6 had a positive PAT (21%) Group 2: 4 had a positive PAT (31%)	NR
Elbarbary et al., 2020^42^	MPFLR	Tegner-Lysholm score: 63	Tegner-Lysholm score: 89.2	All patients had a positive PAT pre-operatively	All patients had a negative PAT post-operatively	No redislocations were reported
Kang et al., 2014^43^	MPFLR	Kujala score: 52.4 Lysholm score: 51.8	Kujala score: 90.9 Lysholm score: 91.7	All patients had a positive PAT pre-operatively	All patients had a negative PAT post-operatively	No redislocations were reported
Niu et al., 2017^26^	MPFLR	Kujala score: 58.9 Lysholm score: 53.3	Kujala score: 92 Lysholm score: 91.6	All patients had a positive PAT pre-operatively	All patients had a negative PAT post-operatively	No redislocations were reported
Chen et al., 2015^22^	Tibial tubercle internal rotation	Kujala score: 56.9 Lysholm score: 51.6	Kujala score: 89.0 Lysholm score: 89.0	All patients had a positive PAT pre-operatively	All patients had a negative PAT post-operatively	No redislocations were reported
Li et al., 2018^17^	Lateral retinacular release and MPFLR	Lysholm score: 68.3	Lysholm score: 92.9	All patients had a positive PAT pre-operatively	All patients had a negative PAT post-operatively	No redislocations were reported
Camathias et al., 2016^44^	Trochleoplasty	Kujala score: 71	Kujala score: 92	Positive PAT in 41 knees (82%)	Positive PAT in 8 knees (16%)	One patella dislocated post-operatively (2%)
Mochizuki et al., 2019^18^	MPFLR	Kujala score: 50.9	Kujala score: 93.7	All patients had a positive PAT pre-operatively	One patient had a positive PAT (4.2%)	No redislocations were reported
Mahmoud et al., 2021^45^	MPFLR	Lysholm score: 59	Lysholm score: 80.2	All patients had a positive PAT pre-operatively	All patients had a negative PAT post-operatively	No redislocations were reported
Kumahashi et al., 2016^46^	MPFLR	Kujala score: 72.2 Lysholm score: 67.8	Kujala score: 96.4 Lysholm score: 96.2	All patients had a positive PAT pre-operatively	One patient had a positive PAT post-operatively (5.9%)	No redislocations were reported
Kumahashi et al., 2012^29^	MPFLR	Kujala score: 67.4 Lysholm score: 64.4	Kujala score: 95.4 Lysholm score: 96.0	All patients had a positive PAT pre-operatively	All patients had a negative PAT post-operatively	No redislocations were reported
Schöttle et al. 2005^47^	MPFLR	Kujala score: 53.3	Kujala score: 85.7	Fourteen knees had a positive PAT (93%)	Three knees remained with a positive PAT (20%)	86.7% reported to recurrent instability
Kita et al., 2012^48^	MPFLR	Kujala score: 73	Kujala score: 95	All patients had a positive PAT pre-operatively	22 knees had a negative PAT (88%)	Patellar maltracking was corrected in all patients

### 3.1 Study quality assessment

The findings of the study quality assessment are presented in Table 1. Of the 34 studies included, 29 were case series. These carry a low level of evidence of 4. Risk of bias of six case series could not be assessed due to these being non-full text studies.^17^^,^^19^, ^20^, ^21^, ^22^, ^23^ Risk of bias was deemed high in one case series due to missing details of patient demographics.^24^ Only three case series carried a low risk of bias.^25^, ^26^, ^27^ There were some concerns regarding the risk of bias in the remaining 19 case series due to their retrospective nature and being performed in a single center.

Five non-randomized comparative studies were included. These comprised a case control study and four cohort studies. The case control study^28^ and two cohort studies^16^^,^^29^ carried some concerns regarding their risk of bias due to lack of blinding of assessors.^28^ Two cohort studies carried a low risk of bias.^9^^,^^30^ Overall, the majority of studies included in this review exhibited methodological limitations in terms of study design and risk of bias (Table 1).

### 3.2 The PAT as a measure of post-operative patella stability

A total of 29 studies utilized the PAT to assess pre- and post-operative patellar stability. Intervention, stability scores, pre- and post-operative PAT and stability outcomes are presented in Table 2. Symptom scores used in the studies included are detailed in Appendix B. Medial patellofemoral ligament reconstruction (MPFLR) comprised the majority of surgical interventions. None of the 29 studies analyzed the relationship between PAT outcomes and patient demographics, anatomical or radiological characteristics. No study reported outcomes in patients with and without a positive PAT separately.

All studies reporting on stability scores reported an improvement from baseline, and a decrease in patients with patellar instability post-operatively. All studies reported a reduction in patients with a positive PAT following surgery aimed at correcting patellar instability. Thirteen studies reported that every patient exhibiting a positive PAT pre-operatively communicated a negative PAT post-operatively. Of the eleven studies which did not, percentage of patients with a positive PAT post-operatively ranged from 4.2% to 42.9%. Five studies reported a high percentage of patients with a positive PAT pre-operatively, instead of the entire population (range: 82%–97%). Number of patients with a positive PAT post-operatively decreased in all of these five studies (range: 11%–21%).

### 3.3 The reliability, validity, and accuracy of the PAT

Ahmad et al. described a new version of the PAT (MPAT) on 51 knees.^9^ The patella is laterally translated while the patient lies supine. The knee is then flexed to 90° and brought back to extension. For the second part, the knee is flexed to 90°, and then extended fully while translating the patella medially. For the MPAT to be considered positive, apprehension must occur during part 1, but not part 2. The MPAT was performed pre-operatively and during knee examination under anaesthesia in 51 patients. The MPAT during examination under anaesthesia was considered the gold standard to diagnose patellar instability. For the detection of patellar instability, the pre-operative MPAT had an accuracy of 94.1%, 100% sensitivity, 88.4% specificity, a negative predictive value of 100%, and a positive predictive value of 89.2%,

Hiemstra et al. measured the inter-rater reliability of the PAT in 38 patients with patellofemoral instability.^31^ Knees were assessed bilaterally by two orthopedic surgeons. The PAT demonstrated fair to substantial reliability (intraclass correlation coefficient was 0.30 for left knees, and 0.65 for right knees). Both surgeons agreed on the PAT status in 27 right knees (71.1%), and 28 left knees (73.7%), indicating moderate to substantial agreement. In addition, evaluation of the PAT in terms of reactions to the test (including verbal, withdrawal, reflex, and physiological) demonstrated good consistency between the two examiners.

Smith et al. assessed the intra- and inter-rater reliability of the PAT.^25^ Five patients were assessed by five consultant Orthopaedic surgeons. The lateral PAT at 0° and 30° demonstrated fair intra-rater reliability (Kappa statistic 0.32 and 0.27, respectively), whereas the medial PAT at 30° knee flexion demonstrated moderate intra-rater reliability (Kappa statistic 0.50). There was slight inter-rater reliability of the PAT at full knee extension (Kappa statistic 0.19), and no inter-rater reliability for the medial and lateral PAT at 30° (Kappa statistic −0.19 and −0.01, respectively).

Zimmermann et al. performed the reversed dynamic PAT (RDPAT) in 78 subjects with recurrent patellar dislocations, and in 35 controls.^30^ During the RDPAT, the patient lies supine, and the knee is brought to extension from 120°, while the patella is translated laterally. The RDPAT is considered positive if apprehension occurs. Seventy-four subjects with recurrent dislocations (94.9%) had a positive RDPAT, whereas 30 control patients (85.7%) had a negative RDPAT. Its specificity was 88.2%, sensitivity 93.7%, negative predictive value was 85.7%, while positive predictive values was 94.9%. Intra- and inter-rater reliability of the RDPAT were 0.85 and 0.84, respectively. In addition, the correlation of a positive RDPAT with patellar instability risk factors was calculated. A positive RDPAT correlated significantly with trochlear dysplasia (p = 0.018) and valgus deformity (p = 0.011). A positive RDPAT did not demonstrate significant correlation with tibial tuberosity – tibial groove (TT – TG) or TT – posterior cruciate ligament (TT – PCL) distances, nor patellar height (p > 0.05).

Hiemstra et al. performed MPFLR in 89 patients to assess the reliability of the PAT.^24^ Apprehension as rated by patient and surgeon were rated on a visual analogue scale (VAS). This was performed at 0° and 30° knee flexion. This was assessed by a single surgeon and his/her patients. Pre-operatively, 81 (91%) and 84 (94.4%) had a positive PAT at 0° and 30° degrees of flexion. This changed to 39 (43.8%) and 36 (40.4%), respectively. The Banff Patellofemoral Instability Score 2.0 (BPII 2.0) improved from a mean of 27.6 before the operation to 74.3 after it. Three subjects experienced a patellar dislocation postoperatively. The surgeon-rated and patient-rated PAT score on the VAS decreased from 5.30 and 6.80 pre-operatively to 1.87 and 2.36, respectively, at 0° knee flexion. At 30°, these decreased from 5.16 and 6.82 pre-operatively to 1.95 and 2.00 as rated by the surgeon and patients, respectively. Pre-operatively, the inter-rater reliability between the surgeon and patients was moderate at 0° (r = 0.60) and weak at 30° (r = 0.42). Post-operatively, there was strong inter-rater reliability in extension (r = 0.74) and 30° flexion (r = 0.73). There was a statistically significant negative correlation between patient-rated apprehension in the VAS and the BPII 2.0 score at 0° knee flexion (r = −0.35, P = 0.001), with less residual apprehension correlating with higher BPII 2.0 scores. This was not the case for 30° of flexion (r = −0.20, P = 0.054). There was no correlation between postoperative patient-rated apprehension in the VAS with ligamentous laxity, patella alta, trochlear dysplasia, WARPS-STAID classification, or age at first dislocation.

## 4 Discussion

A previous systematic review revealed the validity of the PAT was unclear.^10^ This review included studies published since, providing further insight on the validity of this test. Current evidence suggests that the PAT is a valid test to provide a provisional clinical diagnosis of patellar instability. Two studies found it had a high sensitivity, specificity, positive and negative predictive values.^9^^,^^30^ However, these comprised a total of 164 patients. Further study evaluating the accuracy of the PAT are required to ascertain its validity. There is conflicting evidence regarding the intra and inter-rater reliability of the PAT. Four studies assessed these, with results varying widely. Hiemstra et al. found fair to substantial inter-rater reliability,^31^ Zimmermann et al. found strong intra- and inter-rater reliability,^30^ whereas Hiemstra et al. found weak to moderate inter-rater reliability pre-operatively, and a strong reliability post-operatively.^24^ In addition, Smith et al. demonstrated fair to moderate intra-rater reliability pre-operatively, and none to slight inter-rater reliability post-operatively.^25^ Such variability in results can be attributed to differing sample sizes and approaches to performing the PAT. In addition, the PAT is a subjective test, with its results depending heavily on the assessor. This could explain discrepancies in findings between individuals. Therefore, the inter-rater and intra-rater reliability of the PAT are likely to widely vary. Considering this, though the PAT can be used to provide a provisional clinical diagnosis of patellar instability, the PAT alone cannot be used to confirm the diagnosis of recurrent patellar instability. Multiple tests and investigations are required to do so, including functional assessment with validated scoring tools and imaging.

It is not possible to reliably establish if there is an association between a positive PAT and radiological or anatomical features with current evidence. This is because, of the 34 included studies, only two reported on these parameters, and had discrepancies in terms of factors evaluated and findings.^24^^,^^30^ There are insufficient studies exploring the association between a positive PAT and anatomical features. Further research exploring radiological/anatomical features of a positive PAT is required.

Twenty-nine studies utilized the PAT to assess pre- and post-operative patellar stability. All studies reported an increase in stability scores from baseline, and a reduction in subjects exhibiting patellar instability post-operatively. Every study reported a reduced number of subjects communicating a positive PAT post-operatively. This suggests that the PAT is a valid method of assessing patellar stability, since the PAT was negative following surgery aimed at correcting instability. However, no studies reported outcomes separately in patients with and without a positive PAT. In addition, no direct numerical correlation between PAT and stability scores was established, which decreases the validity of this conclusion.

This review identified areas of further research that could further the understanding of the validity of the PAT. Firstly, there is insufficient stratification of outcomes according to anatomical parameters and patient demographics. Exploring the relationship between risk factors for patellar dislocation and PAT outcomes could help determine with more certainty whether this test is a valid method of diagnosing patellar instability. Secondly, no studies comparing the accuracy of the PAT and radiological investigations were identified. The PAT is cheaper given the lack of equipment requirements, and renders an immediate outcome. This is in contrast to imaging, which requires a trained practitioner to perform and evaluate the test, and takes a longer time to convey results. Further research comparing the PAT and radiological investigations could help determine which is a more cost-effective way of diagnosing patellar instability.

Current evidence has limitations that must be improved upon to more reliably ascertain the validity of the PAT. Firstly, there is a lack of a standardized approach to performing the PAT in current literature. It is possible a study's results could be affected if a different variation of the PAT were performed. Furthermore, the lack of a standardized approach to the PAT hinders the generalization of this review's results, since they may not be applicable to all versions of the test. Secondly, no study reported outcomes in patients with and without a positive PAT separately. Therefore, there is a lack of a direct numerical correlation of the PAT to patella stability. This hinders the claim that the PAT is a valid way of assessing patella stability, and impedes the performance of meta-analyses on the subject, which limits the review process. Thirdly, no studies compared the pre-op and post-op sensitivity of the PAT, and only two studies reported the exact point at follow-up in which the PAT was assessed (at one year post-op).^35^^,^^36^ Further study should aim to evaluate these parameters to ascertain whether the PAT is valid both before and after surgery, and to establish the point during follow-up in which the PAT can be reliably used. Finally, the majority of studies included carried a low level of evidence and concerns regarding their risk of bias. This must be taken into consideration when evaluating any conclusions drawn.

## 5 Conclusion

Current evidence suggests that the PAT has a high sensitivity and specificity. The intra- and inter-rater validities of the PAT are widely variable due to its subjective nature. Considering this, though the PAT can be used to provide a provisional diagnosis of patellar instability, the PAT alone cannot be used to confirm the diagnosis of recurrent patellar instability. Multiple tests and investigations are required to do so, including functional assessment with validated scoring tools and imaging. Further research should explore the association between a positive PAT and anatomical parameters under imaging. In addition, studies comparing the accuracy of the PAT and radiological investigations should be performed. The reliability of any conclusions drawn are hindered by limitations of current evidence. These include lack of a standardized approach to performing the PAT, not reporting surgical outcomes according to whether the test result was positive, and the lack of a comparison of PAT validity before and after surgery.

## Funding/sponsorship

This research did not receive any specific grant from funding agencies in the public, commercial or not-for-profit sectors.

## Informed consent

Not applicable.

## Institutional ethical committee approval

Not applicable.

## Author statement

**Diego Agustín Abelleyra Lastoria**: Data curation, Formal analysis, Investigation, Methodology, Visualization, Writing – Original draft preparation, Literature search, Data extraction, Risk of Bias Assessment **Bethany Kenny**: Investigation, Methodology, Literature Search, Risk of Bias Assessment **Sara Dardak**: Conceptualization, Investigation, Methodology, Literature search **Charlotte Brookes**: Conceptualization, Investigation, Methodology, Literature search **Caroline Hing**: Conceptualization, Methodology, Project Administration, Supervision, Writing – Review and Editing.

## Declaration of competing interest

None.

## Figures and Tables

**Table:** 

Parameter	Score
Modified Lysholm limp (5 points)	None = 5. Slightly or periodically = 3. Severe or constant = 0.
Support (5 points)	None = 5. Limp = 2. Weight-bearing impossible = 0.
Locking (15 points)	No locking or crepitation sensation = 15. “Catching” sensation but no locking sensation = 10. Occasional locking = 6. Frequently = 2. Locked joint on examination = 0.
Instability (25 points)	Has never presented buckling = 25. Rarely during sports activities or other exertion = 20. Frequently during sports activities or other exertion = 15. Occasionally in daily living activities = 10. Frequently in daily living activities = 5. With every step = 0.
Pain (25 points)	None = 25. Inconstant and slight during severe exertion = 20. Marked during severe exertion = 15. Marked on or after walking 2 km or more = 10. Marked on or after walking less than 2 km = 5. Constant = 0.
Joint effusion/swelling (10 points)	None = 10. On severe exertion = 6. On slight exertion = 2. Constant = 0.
Stair climbing (10 points)	No problem = 10. Slightly impaired = 6. Step by step (one stair at a time) = 2. Impossible = 0.
Squatting (5 points)	No problem = 5. Slightly impaired = 4. Up to 90° = 2. Impossible = 0.
Total score is out of 100 (100 being the highest possible score):	
Excellent: 91-100	
Good: 84-90	
Fair: 65-83	
Poor: ≤64	

**Table:** 

Parameter	Score
Limp (5 points)	None = 5. Slightly or periodically = 3. Constant = 0.
Support (5 points)	Full support without pain = 5. Painful = 3. Weight-bearing impossible = 0.
Walking (5 points)	Unlimited = 5. More than 2 km = 3. 1–2 km = 2. Unable = 0.
Stairs (10 points)	No difficulty = 10. Slight pain when descending = 8. Pain both when descending and ascending = 5. Unable = 0.
Squatting (5 points)	No difficulty = 5. Repeated squatting painful = 4. Painful each time = 3. Possible with partial weight bearing = 2. Unable = 0.
Running (10 points)	No difficulty = 10. Pain after more than 2 km = 8. Slight pain from start = 6. Severe pain = 3. Unable = 0.
Jumping (10 points)	No difficulty = 10. Slight difficulty = 7. Constant pain = 2. Unable = 0.
Prolonged sitting with the knees flexed (10 points)	No difficulty = 10. Pain after exercise = 8. Constant pain = 6. Pain forces to extend knees temporarily = 4. Unable = 0.
Pain (10 points)	None = 10. Slight and occasional (8). Interferes with sleep = 6. Occasionally severe = 3. Constant and severe = 0.
Swelling (10 points)	None = 10. After severe exertion = 8. After daily activities = 6. Every evening = 4. Constant = 0.
Abnormal painful patellar movements (10 points)	None = 10. Occasionally in sports activities = 6. Occasionally in daily activities = 4. At least one documented dislocation = 2 points. More than two dislocations = 0.
Atrophy of thigh (5 points)	None = 5. Slight = 3. Severe = 0.
Flexion deficiency (5 points)	None = 5. Slight = 3. Severe = 0.
Total score is out of 100 (100 being the highest possible score).	

## Appendix A search strategy

1. Patell*.tw,kf.
2. Patella/
3. Patellofemoral joint/
4. Knee*.tw,kf.
5. Knee joint/
6. 1 or 2 or 3 or 4 or 5
7. (dislocat* or sublux* or instability).tw,kf.
8. Joint Dislocations/
9. Joint Instability/
10. Knee dislocation/
11. Knee Injuries/
12. 7 or 8 or 9 or 10 or 11
13. (apprehension and test).af.
14. *Physical Examination/
15. 13 or 14
16. 6 and 12 and 15
